# Symptoms and Health Complaints and Their Association with Perceived Stressors among Students at Nine Libyan Universities

**DOI:** 10.3390/ijerph111212088

**Published:** 2014-11-25

**Authors:** Walid El Ansari, Khalid Khalil, Christiane Stock

**Affiliations:** 1 Faculty of Applied Sciences, University of Gloucestershire, Gloucester GL2 9HW, UK; 2 Faculty of Medical Technology, Misrata, Libya; E-Mail: khalid8128@yahoo.com; 3 Unit for Health Promotion Research, Institute of Public Health, University of Southern Denmark, Esbjerg 6700, Denmark; E-Mail: cstock@health.sdu.dk

**Keywords:** self-reported symptoms/ health complaints, stressors, burdens, quality of life, university students

## Abstract

University students are exposed to many stressors. We assessed the associations between two stressors (educational related and general overall), socio-demographic characteristics (five variables), health behaviours/lifestyle factors (six variables), as well as religiosity and quality of life as independent variables, with self-reported symptoms/health complaints as dependent variables (eight health complaints). A sample of 2100 undergraduate students from nine institutions (six universities, three colleges) located in seven cities in Libya completed a general health questionnaire. The most prevalent symptoms were headaches, depressive mood, difficulties to concentrate and sleep disorder/insomnia that have been reported by 50%–60% of the students. The majority of students (62%) reported having had three or more symptoms sometimes or very often in the last 12 months. There was a positive association between perceived stressors and health symptoms, which remained significant after adjustment for gender and many other relevant factors for headache (OR 1.52; 95% CI 1.15–2.02), depressive mood (OR 2.20; 95% CI 1.64–2.94) and sleep disorder/ insomnia (OR 1.55, 95% CI 1.19–2.03). Other factors independently associated with most health symptoms were female gender and poor self-perceived health. Stress management programmes and a reduction of educational related stressors might help to prevent stress-related symptoms and health complaints in this student population.

## 1. Introduction

There is an increased focus on the health and well-being of students at higher education institutions, and calls that is “time for urgent action” for the health of these young adults [1]. In particular, educators are concerned about the sources of stress (stressors or burdens) amongst university students [2,3]. Stressors are “demands made by the internal or external environmental stimuli that affect the balance, thus influencing physical and psychological well-being of an individual and requiring actions to restore the balance” [4]. Whilst it is argued that stressors might beneficially motivate students’ achievement, performance, enhanced productivity and reward [5], their negative effects include self-reported symptoms, health complaints, psycho-somatic symptoms, or health strains.

Such symptoms and complaints might be categorized into four groups: physical features (back pains, neck/ shoulder pains, headaches); gastrointestinal symptoms (stomach trouble/heartburn); psychological, psychiatric and mental health issues (depression, sleep disturbances, difficulties to concentrate); and circulatory complaints (rapid heartbeats, circulatory problems, dizziness) as manifestations of or associated with a range of stressors [2,6]. Students might be overwhelmed by their university experience to an extent that their physical and mental health could be negatively affected [7]. For example, perceived stressors related to studying were positively associated with higher depression among students from Germany, Poland and Bulgaria, by mediation via perceived stress and also directly [8].

The sources of such burdens/stressors for college students are many. One important aspect is the education or curricular (university study-related) stressors. Academic demands are considerable stressors [9], and the university period could be a stressor for students trying to achieve academic success despite financial constraints [10]. College students’ major stressors due to examinations suggested the use of reported symptoms for early detection of stress and proper intervention [11]. Stressors include personal expectations, peer competition, having to attain good grades, or fear of failing/repeating their course [12]. In Colombia, “fear of failing a course or year” was the highest ranked item, seen as very stressful by >60% of the sample, while “examinations and grades” ranked the third highest [13]. Other stressors/burdens are related to the general social atmosphere/environment of the students; e.g., being away from home, new socializations [12], and financial pressures [14].

Unsurprisingly, university students in many countries report educational-related and general stressors e.g., in Canada, UK, Egypt, Japan, Iran, and Jordan [2,6,15,16,17,18]. Similarly, university students across the globe report many symptoms/health complaints: in Japan, >40% of students had headache, stomach ache/abdominal pain, and stiff shoulder/backache within the past month [16]; and in the UK and Egypt, health complaints that occurred most often in the last year were fatigue, headache, difficulties to concentrate, back pain, neck/shoulder pain, and sleep disorders [2,6].

The stressors that students face and the accompanying health symptoms are an increasing problem that adversely affect their health. We investigated two types of stressors: college-related stressors; and general life stressors [19]. Better understanding of students’ symptoms (health complaints) and (educational or general) stressors and their frequencies, along with the associations between health complaints and students’ demographics and health behaviours, and the associations between individual symptoms and stressors are important to tailor effective interventions. Few studies of stressors and associated symptoms of university students have been conducted in some Eastern Mediterranean countries, and no such research has been implemented in Libya. Given this scarcity, together with challenges/ barriers imposed by the previous political regime in terms of accessing universities within Libya, such young adult college populations are hard-to-reach. The current research bridges these knowledge gaps, attaching high significance to the study’ contributions and findings.

### Aim of the Study

This cross-sectional survey of a representative sample of undergraduate students across nine Libyan universities/ colleges (2008–2009) assessed the frequencies of self-reported students’ symptoms (eight health complaints) and two stressors (educational related and general overall). We also examined the associations between the health complaints and students’ socio-demographics (five variables—age, marital status, year of study, living arrangement during semester, income sufficiency) and health behaviours/lifestyle features (six variables—smoking, illicit drug/s, alcohol, subjective health, health awareness, BMI), as well as religiosity and quality of life. The study also explored the associations between the four most prevalent symptoms and stressors. The four specific objectives were to:

- Describe the sample’s general characteristics;
- Assess the prevalence of eight symptoms, and the number of symptoms reported in the last 12 months;
- Assess the association between the frequency of the four most prevalent symptoms and students’ demographic characteristics and health behaviours; and
- Explore the frequency of symptoms by extent of perceived stressors (burdens); and the association between the four most prevalent symptoms and perceived stressors while controlling for all other symptom groups.

## 2. Materials and Methods

### 2.1. Sample, Data Collection and Ethics

The study was approved by research/ethics committees at the participating institutions. A representative sample of students was sought at the universities by selecting courses that represented the different departments/faculties. Questionnaires (self-administered) were distributed to students attending the selected sessions, and collected after completion. Participation was voluntary and anonymous, and data were confidential and protected. All data were computer-entered by the second author for quality assurance. The questionnaire was provided to 2100 students, and 1567 completed questionnaires were returned (response rate ≈ 74.6%), of which 267 questionnaires were excluded (missing data), leaving 1300 questionnaires for analysis (439 males, 33.8%; 861 females, 66.2%; M age 20.9 years, SD 2.4). Nine institutions (six universities, three colleges) in seven cities (Misurata, Sabha, Zawea, Sirt, Al Bida, Benghazi, Tripoli) participated, thus the sample was representative of Libyan higher education institutions and of many scientific/academic disciplines: e.g., Agriculture, Business, Education, Law, Mechanical Engineering, Medical Science, Medical Technology, and others. In Libya (academic year 2010/2011), more than 90% of the university students were enrolled in public universities (59% females). Thus the current study included most public universities in order to ensure representativeness of the study sample to the greater population of university students in Libya. In addition, geographically, the universities that were included in the study were situated across Libya, in the north, west, east and south, also ensuring representativeness of the study sample.

Generally in Libya, Higher Education is completely financed by the state, except for private universities. All high school students have the access to higher education institutions. Every year, the Cabinet determines the regulations for students’ admission to higher education institutions by considering the results of the “Secondary School Leaving Certificate”, the needs of society, and institutions’ capacities. Students’ fees are very small; students pay only registration fees at the beginning of the academic year or semester. In terms of the economic situation, until recently Libya was classified as a high-income country, but has lately moved to low-income country status.

### 2.2. Health and Wellbeing Measures and Variables

This general student health survey [2,3,4,6,20,21,22,23,24,25,26,27] utilized a questionnaire that included general health/wellbeing information, perceived stressors (burdens), and nine symptoms/health complaints. The data comprised gender, age, marital status, year of study, living arrangements (during semester), smoking, alcohol consumption, subjective health status, health awareness, height and weight (to compute BMI), importance of religion/personal faith (religiosity), and income sufficiency.

*Perceived Stressors* (burdens) (two items): one item focused on students’ educational/university related burdens (course work, exams): “To what extent do you feel burdened in the following areas?”. The second item assessed general burdens overall. “Considering your current situation, to what extent do you feel burdened overall?” [8,28].

*Health problems, symptoms/ health complaints* (eight items): students rated eight symptoms/health complaints [2,3,29]. “How often have you had these complaints during the past 12 months?” (1 = “never”; 4 = “very often”), e.g., stomach trouble/heartburn, back pain, rapid heart beats/circulatory problem/dizziness, headaches, sleep disorder/ insomnia, concentration difficulties, neck and shoulder pain, and depressive mood. In our sample, Cronbach’s alpha (whole scale) was 0.74.

*Marital status*: “What is your marital status?” (“Married”, and “Single”).

*Living arrangements* during semester time: “Where do you live (during university/college term time)?” with two options based on whether the participant was living with parents or not.

*Tobacco smoking*: “Within the last three months, how often did you smoke? (cigarettes, pipes, cigarillos, cigars)” (“daily”, “occasionally”, “never”) [30].

*Illicit drug/s use*: “Have you ever use/used drugs?” (“Yes, regularly”, “Yes, but only a few times”, “Never”), recoded into two options based on whether the participant ever used illicit drug/s or not [24].

*Alcohol consumption frequency*: “Over the past three months how often have you drunk alcohol, e.g., beer?” (six options: “never”, “once a week or less”, “once a week”, “a few times each week”, “every day”, and “a few times each day”).

*Subjective health status*: “How would you rate your health in general?” (1 = “excellent”, 5 = “poor”) [31].

*Health awareness*: “To what extent do you keep an eye on your health?” (1 = “not at all”, 4 = “very much”) [28].

*BMI (reported)*: calculated from self-reported weight and height using Metric BMI Formula (BMI (kg/m^2^) = weight in kilograms/squared height (m^2^)), and categorised into: underweight (BMI < 18.5 kg/m^2^), normal (18.5 ≤ BMI ≤ 24.9 kg/m^2^), overweight (25.0 ≤ BMI ≤ 29.9 kg/m^2^), or obese (BMI ≥ 30.0 kg/m^2^) [32].

*Importance of religion*/*personal faith* (religiosity: “My religion is very important for my life?” (1= “strongly disagree”, 5= “strongly agree”), recoded into two options based on whether the participant agreed or not.

*Income sufficiency* (subjective economic situation): how sufficient students considered the amount of money they have at their disposal (4-point scale: “always sufficient”, “mostly sufficient”, “mostly insufficient”, “always insufficient”).

*Quality of one’s life*: “If you consider the quality of your life: How did things go for you in the last four weeks?” (1 = “very badly”, 5 = “very well”) [33], later recoded into three categories.

### 2.3. Statistical Analysis

The Statistical Package SPSS v19.0 was used for the statistical analyses (*p* set at <0.05). For descriptive analyses, categorical data were expressed as frequencies and percentages, and score data presented as means and standard deviations. Chi-Square tests compared the categorical variables between males and females. For each symptom, two-sample *t*-test or ANOVA compared the mean levels of symptoms across variables of interest (e.g., year of study, age, marital status *etc.*). We employed Bonferroni adjustment for multiple testing for the statistical comparisons (*p*-value set at *p* ≤ 0.001). Linear regression assessed the association between increasing levels of feeling burdened overall and increasing frequency of symptoms (*p* set at <0.05). Multifactorial logistic regression analysed the relationship between general stressor (feeling burdened overall strongly/very strongly), other students’ general characteristics as independent variables, and each of the four most prevalent symptoms, each as a dependent variable. Only the variables significant in initial bivariate tests were included in the final model. Odds ratios were adjusted for all variables in the models.

## 3. Results

### 3.1. General Characteristics

The sample comprised 66% females, 65% were 20–24 years old, 97% not married, 82% living with their parents, and 98% reported that religion was of high importance in their life, while 73% regarded their income as mostly or always sufficient (Table 1).

**Table 1:** General characteristics of the sample.

Variable	Characteristic	All Students N = 1300	Males N = 439	Females N = 861	*p*
**Stressors**					
Burdens (exams)	Less burdened	573 (44.1)	243 (55.4)	330 (38.3)	**<0.001**
	Strongly/Very strongly	727 (55.9)	196 (44.6)	531 (61.7)	
Burdens (overall)	Less burdened	972 (74.8)	338 (77.0)	634 (73.6)	0.187
	Strongly/Very strongly	328 (25.2)	101 (23.0)	227 (26.4)	
**Socio-demographic**					
Age (years)	<20	360 (27.7)	109 (24.8)	251 (29.2)	
	20–24	848 (65.2)	288 (65.6)	560 (65.0)	**0.020**
	≥25	92 (7.1)	42 (9.6)	50 (5.8)	
Marital status	Married	45 (3.5)	5 (1.1)	40 (4.6)	**0.001**
	Single	1255 (96.5)	434 (98.9)	821 (95.4)	
Year of study	1st	431 (33.2)	187 (42.6)	244 (28.3)	**<0.001**
	2nd	356 (27.4)	86 (19.6)	270 (31.4)	
	3rd	319 (24.5)	82 (18.7)	237 (27.5)	
	≥4th	194 (14.9)	84 (19.1)	110 (12.8)	
Living with parent	Yes	1062 (81.7)	322 (73.3)	121 (14.1)	
	No	238 (18.3)	117 (26.7)	740 (85.9)	**<0.001**
Income sufficiency	Always/Mostly sufficient	948 (72.9)	285 (64.9)	663 (77.0)	**<0.001**
	Mostly/Always insufficient	352 (27.1)	154 (35.1)	198 (23.9)	
**Health behaviours/Lifestyle**					
Smoking	Daily	63 (4.8)	63 (14.4)	0	
	Occasional	49 (3.8)	43 (9.8)	6 (0.7)	**<0.001**
	Never	1188 (91.4)	333 (75.9)	855 (99.3)	
Illicit drug/s (ever use)	No	1279 (98.4)	419 (95.4)	860 (99.9)	**<0.001**
	Yes	21 (1.6)	20 (4.6)	1 (0.1)	
Alcohol consumption	Never	1256 (96.6)	404 (92.0)	852 (99.0)	
	Occasional	40 (3.1)	31 (7.1)	9 (1.0)	**<0.001**
	Every day	4 (0.3)	4 (0.9)	-	
Subjective health	Excellent/Very good	690 (53.1)	228 (51.9)	462 (53.7)	
	Good	424 (32.6)	149 (33.9)	275 (31.9)	0.765
	Fair/Poor	186 (14.3)	62 (14.1)	124 (14.4)	
Health awareness	Very much/To some extent	1043 (80.2)	364 (82.9)	679 (78.9)	0.083
	Not much/Not at all	257 (19.8)	75 (17.1)	182 (21.2)	
BMI (reported) ^*^	Underweight	86 (8.2)	24 (6.1)	62 (9.4)	
	Normal weight	667(63.5)	228 (58.0)	439 (66.8)	**<0.001**
	Overweight	236 (22.5)	106 (27.0)	130 (19.8)	
	Obese	61 (5.8)	35 (8.9)	26 (4.0)	
**Others**					
Importance of religion	Somewhat/Strongly disagree	22 (1.7)	12 (2.8)	10 (1.2)	**0.035**
(religiosity)	Strongly/Somewhat agree	1269 (98.3)	420 (97.2)	849 (98.8)	
Quality of life	Very badly/Badly	116 (8.9)	45 (10.3)	71 (8.5)	0.439
	Intermediate	416 (32.0)	142 (32.3)	274 (31.8)	
	Quite well/Very well	768 (59.1)	252 (57.4)	516 (59.9)	
**Total**		1300 (100)	439 (100)	861 (100)	

* Calculated based on self-reported height and weight according to WHO guidelines—underweight (BMI < 18.5 kg/m^2^), normal weight (BMI of 18.5–24.9 kg/m^2^), overweight (BMI of 25.0–29.9 kg/m^2^), or obese (BMI ≥ 30.0 kg/m^2^) [32].

Most respondents (80%) watched their health to some extent or very much (health awareness), 53% perceived their health as very good or excellent, 59% rated their quality of life as quite well or very well, but 28% were overweight or obese based on their BMI. There was a low proportion of smokers (9%), those who had ever used illicit drug/s (2%) or reported occasional/daily alcohol (3%) use. More than half (56%) the sample felt strongly/very strongly burdened by exams, and one quarter felt strongly/ very strongly burdened overall. Compared with males, female students were younger, more often married, had more often insufficient income, were enrolled in higher years study, lived more often with parents, consumed alcohol or other illict drug/s less frequently, were less often overweight or obese, and felt more burdened by exams.

### 3.2. Prevalence and Number of Symptoms in Last 12 Months

About 50%–60% of the sample had headaches, depressive mood, difficulties to concentrate or sleep disorder/insomnia sometimes/very often during the last year (Table 2). The prevalence of these four symptoms was highest, followed by back pain and shoulder and neck pain. Less than one third of students had circulatory problems or stomach trouble/heartburn sometimes or very often.

**Table 2:** Prevalence of symptoms during last 12 months.

Symptoms	Never	Rarely	Sometimes/ Very Often
	N (%)	N (%)	N (%)
**Psychological**			
Depressive mood	306 (23.5)	245 (18.8)	749 (57.6)
Difficulties to concentrate	227 (17.5)	335 (25.8)	738 (56.8)
Sleep disorder/Insomnia	361 (27.8)	290 (22.3)	649 (49.9)
**Circulatory**			
Rapid heartbeats, Circulatory problems, Dizziness	718 (55.2)	245 (18.8)	337 (25.9)
**Pains/Aches**			
Back pain	391 (30.1)	319 (24.5)	590 (45.4)
Neck and shoulder pain	492 (37.8)	321 (24.7)	487 (37.5)
Headaches	172 (13.2)	340 (26.2)	788 (60.6)
**Gastrointestinal**			
Stomach trouble/Heartburn	618 (47.5)	265 (20.4)	417 (32.1)

All percentages are row percentages rounded to one decimal point.

Table 3 depicts the proportion of students with no symptoms, 1–2 or 3 or more symptoms. A majority of students (62%) and significantly more females than males reported having had ≥3 symptoms, while only 8% had no symptom (sometimes or very often) during the last year.

**Table 3:** Number of symptoms reported in last 12 months.

Sample	No Symptoms	1–2 Symptoms	≥3 Symptoms	*p* value for Gender Difference
	N (%)	N (%)	N (%)	
**All students**	97 (7.5)	394 (30.3)	809 (62.2)	
**Males**	42 (9.6)	152 (34.6)	245 (55.8)	**0.002**
**Females**	55 (6.4)	242 (28.1)	564 (65.5)	

All symptoms counted if reported to occur Sometimes or Very often.

### 3.3. Frequency of Symptoms by General Characteristics

Table 4 depicts the frequency of the four most prevalent symptoms expressed as mean rating (1 = “never”, 4 = “very often”) by students’ general characteristics and health behaviours. After Bonferroni adjustment, some associations were significant. Participants perceiving their health as fair/poor, those who were strongly/very strongly burdened by exams, and those who were strongly/very strongly burdened overall had consistently significantly higher ratings across all the four most prevalent symptoms when compared with those reporting very good/excellent health or feeling less burdened.

**Table 4:** Frequency of symptoms by general characteristics and by health behaviour.

Characteristic/ Behaviour	Headache *M* (SD)	*p*	Depressive Mood *M* (SD)	*p*	Difficulties to Concentrate *M* (SD)	*p*	Insomnia *M* (SD)	*p*
***Stressors***								
**Stressors/Burdens (exams)**								
Less burdened	2.52 (0.92)	**<0.001**	2.31 (1.07)	**<0.001**	2.33 (0.94)	**<0.001**	2.20 (1.05)	**<0.001**
Strongly/very burdened	2.78 (0.93)		2.80 (1.07)		2.71 (0.92)		2.55 (1.06)	
**Stressors/Burdens (overall)**								
Less burdened	2.59 (0.91)	**<0.001**	2.43 (1.06)	**<0.001**	2.46 (0.92)	**<0.001**	2.29 (1.04)	**<0.001**
Strongly/very burdened	2.88 (0.99)		3.01 (1.07)		2.77 (0.99)		2.69 (1.10)	
***Socio-demographic***								
**Gender**								
Female	2.76 (0.92)	**<0.001**	2.69 (1.09)	**<0.001**	2.58 (0.93)	0.031	2.47 (1.06)	**0.001**
Male	2.48 (0.93)		2.36 (1.06)		2.46 (0.97)		2.25 (1.07)	
**Age**								
< 20	2.57 (0.90)	0.050	2.45 (1.08)	0.006	2.43 (0.94)	0.018	2.17 (1.05)	**<0.001**
20-24	2.71 (0.94)		2.61 (1.10)		2.57 (0.95)		2.47 (1.07)	
≥ 25	2.60 (1.02)		2.83 (1.06)		2.68 (0.88)		2.60 (1.02)	
**Marital status**								
Married	2.69 (0.93)	0.868	2.13 (1.08)	0.005	2.29 (0.92)	0.068	2.38 (1.03)	0.914
Single	2.67 (0.94)		2.60 (1.09)		2.55 (0.95)		2.40 (1.07)	
**Year of study**								
1st	2.56 (0.91)	0.007	2.42 (1.10)	**0.001**	2.43 (0.96)	0.034	2.18 (1.04)	**<0.001**
2nd	2.68 (0.95)		2.58 (1.10)		2.59 (0.94)		2.46 (1.08)	
3rd	2.80 (0.92)		2.73 (1.08)		2.58 (0.91)		2.56 (1.05)	
≥4th	2.65 (0.94)		2.66 (1.06)		2.63 (0.98)		2.49 (1.07)	
**Living with parent**								
Yes	2.69 (0.92)	0.041	2.58 (1.09)	0.998	2.55 (0.93)	0.656	2.39 (1.07)	0.635
No	2.55 (0.97)		2.58 (1.10)		2.52 (1.01)		2.42 (1.08)	
**Income sufficiency**								
Always/ Mostly sufficient	2.66 (0.93)	0.482	2.52 (1.10)	0.002	2.49 (0.95)	**0.001**	2.36 (1.06)	0.089
Mostly/ Always insufficient	2.70 (0.95)		2.73 (1.07)		2.68 (0.93)		2.48 (1.09)	
**Health behaviours/Lifestyle**								
**Smoking**								
Daily	2.63 (0.97)	0.069	2.62 (1.04)	0.089	2.57 (1.00)	0.590	2.40 (1.06)	0.902
Occasional	2.37 (1.06)		2.24 (1.11)		2.41 (0.93)		2.33 (1.11)	
Never	2.68 (0.93)		2.59 (1.09)		2.55 (0.94)		2.40 (1.07)	
**Illicit drugs** (ever use)								
No	2.66 (0.93)	0.636	2.58 (1.09)	0.869	2.54 (0.94)	0.541	2.39 (1.07)	0.240
Yes	2.76 (1.04)		2.62 (1.28)		2.67 (1.16)		2.67 (1.07)	
**Alcohol consumption**								
Never	2.67 (0.93)	0.888	2.58 (1.09)	0.668	2.54 (0.94)	0.837	2.39 (1.07)	0.756
Occasionally	2.60 (0.98)		2.50 (1.20)		2.60 (1.08)		2.45 (1.04)	
Every day	2.75 (1.50)		3.00 (1.41)		2.75 (1.26)		2.75 (0.96)	
**Subjective Health**								
Excellent/Very good	2.51 (0.92)	**<0.001**	2.41 (1.10)	**<0.001**	2.38 (0.94)	**<0.001**	2.23 (1.05)	**<0.001**
Good	2.82 (0.88)		2.65 (1.04)		2.63 (0.89)		2.51 (1.03)	
Fair Poor	2.89 (0.99)		3.04 (1.04)		2.93 (0.95)		2.73 (1.13)	
**Health awareness**								
Very much/Some extent	2.63 (0.93)	0.014	2.51 (1.08)	**<0.001**	2.49 (0.92)	**<0.001**	2.37 (1.07)	0.054
Not much/Not at all	2.79 (0.96)		2.86 (1.09)		2.75 (1.02)		2.51 (1.07)	
**BMI (reported)**								
Underweight	2.60 (0.92)	0.620	2.58 (1.15)	0.313	2.44 (0.94)	0.339	2.29 (1.11)	0.695
Normal weight	2.63 (0.91)		2.62 (1.08)		2.57 (0.92)		2.42 (1.06)	
Overweight	2.69 (0.91)		2.47 (1.08)		2.50 (0.93)		2.43 (1.04)	
Obese	2.74 (1.06)		2.64 (1.13)		2.67 (1.00)		2.36 (1.07)	
**Others**								
**Importance of religion** (religiosity)								
Somewhat/Strongly disagree	2.73 (0.88)	0.766	2.82 (1.05)	0.309	2.55 (0.96)	0.984	2.32 (0.89)	0.721
Strongly/Somewhat agree	2.67 (0.94 )		2.58 (1.09)		2.54 (0.95)		2.40 (1.07)	
**Quality of life**								
Very badly/Badly	2.74 (0.97)	0.412	2.92 (1.05)	**<0.001**	2.80 (0.93)	**<0.001**	2.64 (1.17)	**<0.001**
Intermediate	2.69 (0.97)		2.77 (1.05)		2.68 (0.95)		2.52 (1.04)	
Quite well/Very well	2.64 (0.91)		2.42 (1.09)		2.43 (0.93)		2.29 (1.06)	

SD rounded to one decimal point; All *P* values based on *t*-test or ANOVA; Significance level after Bonferoni adjustment set at *p* = 0.001; Symptoms measured on a four-point response scale, 1 = “never”; 4 = “very often”.

Across all four symptoms (except for difficulties to concentrate), females had higher ratings of complaints than males. For quality of life, those feeling bad/very bad quality of life had consistently significantly higher ratings of symptoms (except for headache). Students who watched their health not much/not at all (lower health awareness) had significantly higher ratings of depressive mood and difficulties to concentrate.

Students reported increasing frequency of insomnia with older age, and increasing frequency of depressive mood and insomnia with higher year of study. Those with insufficient income had significantly higher difficulties to concentrate than students who had always/ mostly sufficient income. The associations between symptoms and all the remaining variables did not reach statistical significance (according to the Bonferroni adjusted level of *p* = 0.001).

### 3.4. Association between Feeling Burdened Overall and Frequency of Symptoms

**Figure 1:**
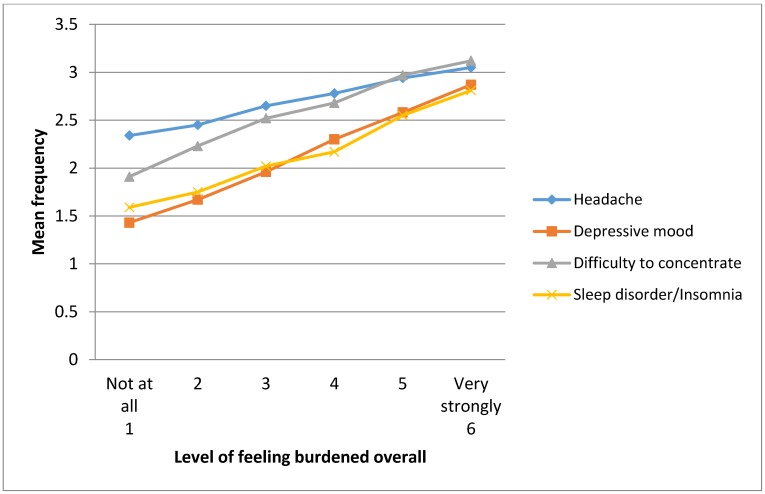
Mean frequency of headache, insomnia, difficulties to concentrate and headache by level of feeling burdened overall.

Table 5 shows the frequency of the four most prevalent symptoms expressed as mean rating (from 1= “never”, 4 = “very often”) by increasing level of feeling generally burdened overall (6-point Likert scale from “not at all” to “very much”). A significant linear regression between the frequency of symptoms and the level of feeling burdened overall was observed for all four symptoms, but the regression coefficient was highest for depressive mood. Figure 1 depicts a positive relationship of increasing frequency of symptoms with increasing levels of feeling generally burdened overall.

**Table 5:** Mean frequency of symptoms by level of feeling burdened overall.

Symptom	Level of Stressor (Feeling Burdened)							*p*
	Not at All					Very Much	β	
	M (SD)	M (SD)	M (SD)	M (SD)	M (SD)	M (SD)		
Headache	2.34(0.83)	2.45(0.84)	2.65(0.82)	2.78(0.83)	2.94(0.82)	3.05(0.91)	0.18	<0.001
Depressive mood	1.43(0.69)	1.67(0.75)	1.96(0.85)	2.30(0.89)	2.58(0.92)	2.87(0.96)	0.30	<0.001
Difficulty to concentrate	1.91(0.91)	2.23(0.85)	2.52(0.79)	2.68(0.82)	2.97(0.79)	3.12(0.86)	0.20	<0.001
Sleep disorder/ insomnia	1.59(0.86)	1.75(0.93)	2.02(0.97)	2.17(0.98)	2.55(1.08)	2.81(1.06)	0.19	<0.001

Symptoms measured on a four-point response scale (1 = “never”; 4 = “very often”); β coefficients and *p*-values based on linear regression.

Table 6 shows the associations between feeling generally burdened overall (strongly/very strongly) and having experienced each of the four most prevalent symptoms sometimes or very often as dependent variables while adjusting for a number of general characteristics. After adjustment, students feeling burdened strongly or very strongly were significantly more likely to suffer headache, depressive mood and sleeping disorder/insomnia, but not difficulty to concentrate.

Across all symptoms, perceived (subjective) poor health was consistently associated with higher frequency of all four symptoms. Students who watched their health not much/not at all (lower health awareness) were more likely to report depressive mood. Better quality of life was associated with lower frequency of depressive mood. Females were more likely to have headache, depressive mood, and sleeping problems/insomnia than the males. Students were more likely to have difficulty to concentrate when their income was always/mostly insufficient. Older students had more sleep disorder/insomnia, and students in higher study year had more depressive mood.

**Table 6:** Adjusted odds ratios for associations between symptoms and feeling burdened overall and with general characteristics.

Variable	Headache OR (95% CI)	Depressive Mood OR (95% CI)	Difficulty to Concentrate OR (95% CI)	Sleep Disorder/ Insomnia OR (95% CI)
**Stressors**				
**Feeling burdened overall**				
Less burdened	1.00	1.00	1.00	1.00
Strongly/very strongly burdened	**1.52** (1.15–2.02)	**2.20** (1.64–2.94)	1.07 (0.81–1.40)	**1.55** (1.19–2.03)
**Socio-demographic**				
**Gender**				
Female	1.00	1.00	1.00	1.00
Male	**0.63** (0.49–0.80)	**0.58** (0.45–0.75)	1.12 (0.87–1.44)	0.71 (0.56–0.91)
**Age**				
<20	1.00	1.00	1.00	1.00
20-24	1.22 (0.90–1.63)	1.07 (0.79–1.44)	1.01 (0.74–1.36)	1.46 (1.09–1.96)
≥ 25	0.88 (0.52–1.48)	1.32 (0.76–2.29)	1.13 (0.66–1.93)	**1.92** (1.14–3.26)
**Year of study**				
1st *	1.00	1.00	1.00	1.00
2nd	1.16 (0.86–1.58)	1.07 (0.78–1.46)	0.89 (0.65–1.21)	1.25 (0.92–1.69)
3rd	1.13 (0.80–1.60)	1.34 (0.94–1.90)	1.00 (0.70–1.41)	1.37 (0.98–1.92)
≥4th	1.05 (0.70–1.57)	**1.68** (1.11–2.53)	1.08 (0.72–1.62)	1.36 (0.92–2.01)
**Income sufficiency**				
Always/Mostly sufficient	1.00	1.00	1.00	1.00
Always/Mostly insufficient	1.05 (0.81–1.37)	1.29 (0.98–1.69)	**1.33** (1.02–1.73)	1.05 (0.81–1.36)
**Health behaviours/Lifestyle**				
**Subjective health status**				
Excellent/Very good	1.00	1.00	1.00	1.00
Good	**1.64** (1.27–2.13)	1.21 (0.93–1.56)	1.31 (1.01–1.70)	1.35 (1.05–1.73)
Fair/Poor	1.57 (1.09–2.27)	**1.63** (1.10–2.41)	**2.39** (1.67–3.41)	1.51 (1.06–2.17)
**Watch one’s health**				
Very much/Some extent	1.00	1.00	1.00	1.00
Not much/Not at all	0.97 (0.72–1.30)	1.41 (1.03–1.92)	1.16 (0.87–1.56)	1.04 (0.78–1.39)
**Others**				
**Quality of life**				
Very badly/Badly	1.00	1.00	1.00	1.00
Intermediate	0.84 (0.54–1.31)	0.89 (0.55–1.43)	0.97 (0.63–1.48)	1.06 (0.69–1.63)
Quite well/Very well	1.08 (0.70–1.67)	**0.60** (0.38–0.95)	0.66 (0.44–1.01)	0.86 (0.57–1.31)

OR: odds ratio (adjusted for the other three groups of symptoms); 95% CI: confidence interval; **Bolded** cells indicate statistical significance (at least *p* < 0.05).

## 4. Discussion

University students are subjected to stressors and they respond to it on daily basis. Researchers have also acknowledged the stressful nature of the students’ roles and expectations [34]. The current study bridges the knowledge gaps to assess, across a representative sample of students at nine different universities in Libya, students’ self-reported symptoms (health complaints) and (college-related and general) stressors, and their frequencies. We also scrutinized the associations between the health complaints and students’ demographic characteristics and health behaviours, as well as the associations between each symptom and stressors adjusting for a number of other relevant factors. To the best of our knowledge, this is the first published in depth examination of such issues in Libya.

As for the study’s first objective, there were more females, and the majority of students were single, living with their parents, and religion was of high importance in their lives. These features need to be considered within a range of issues of Libya’s geopolitical situation, prevalent cultural norms, and prevailing religious Islamic faith. Across most Arabic Eastern Mediterranean countries generally, it is quite traditional for unmarried individuals/ students to live with their parents, particularly for females. Likewise, our finding of the high importance to religiosity needs to be viewed in the context that Libya is of predominantly Muslim faith, where it is customary to pray five times each day, and for public modesty, many/most women might wear loose traditional dress (abayas) covering the body and not revealing the silhouette, or at least a Hijab for their heads concealing the hair and neck. Similarly, the higher proportion of females in the Libyan sample reflects what seems to be a reality at higher education institutions globally, and is in agreement with the gender distributions of university student samples from other higher income countries e.g., England, Wales and Northern Ireland, Sweden, Ireland, Korea [27,35,36,37], or lower income countries e.g., Colombia or Egypt [2,38]. We also agree with Iran, where across 300 students, 91% were single 71% lived in dormitories, 26% lived at home with their families and only 3% lived alone in rental properties [17].

In terms of objective two, our sample reported headaches (60.6%), difficulties to concentrate (56.8%), depressive mood (57.6%), sleep disorder/ insomnia (49.9%) as the four symptoms that most often occurred sometimes/very often in the last year. The other remaining symptoms appeared to various extents, and were reported as sometimes/ very often by about one third to one quarter of the students. The array of symptoms across our sample agrees with findings of students from seven European countries [29], where headache, back ache, and neck/shoulder pain were often reported complaints, suggesting a prevailing role of headache among self reported symptoms of college students. Our findings also agree with and are quite similar to levels of health complaints reported by students across seven universities in England, Northern Ireland and Wales, where symptoms that occurred sometimes/very often in the last year included headache (59.5%) and difficulties to concentrate (54.4%) [6]. The UK study [6] also reported back pain (43.3%) and neck/shoulder pain (39.4%), both of which were comparable to our Libyan sample (45.4% back pain; 37.5% neck/shoulder pain). However, the Libyan levels were lower than those of Egypt, where the health complaints that occurred sometimes/very often in the last year included difficulties to concentrate (78.1%), headache (77.9%), and sleep disorders (63.7%) [2]. It is unlikely that the higher health complaints levels in Egypt [2] than those in Libya were related to methodological issues, as the same research instrument was employed in both countries (although the symptoms list used in Libya had slightly less items). It remains speculative why the levels in Egypt were higher than in Libya, although both these eastern Mediterranean neighbouring countries share similarities (history, traditions, culture, religion and context), as well as contrasts. Other similarities between Libya and Egypt, as observed in Oman, is that most students in these Middle Eastern countries who join university leave their homes for the first time (loss of the traditional social support and supervision), cohabite with other students (peer relationships), and face alterations in the learning styles from what students were acquainted with at school [39]. Hence, it is unclear if these observed differences actually reflect real differences in the prevalence of complaints, or if specific cultural factors might contribute to higher “readiness” to report health complaints among Egyptian students compared to their Libyan peers.

As for the number of symptoms reported in the last year, ≈62% of the Libyan sample and significantly more females than males reported having had ≥3 symptoms, while only 8% had no symptom (sometimes/very often) during the last year. One might conclude that experiencing at least one health complaint can be considered as a normal condition in this population of young adults, a finding that is in line with research in Egypt [2].

In connection with objective three, females generally reported significantly higher frequency across all four symptoms, in agreement with the higher levels of health complaints among female university students [28,40]. In Jordan, there were statistical differences between male and female students regarding their perception and reactions to stressors [18]. Women might report higher levels of health problems because of reduced access to resources and social conditions of life that foster health, and because of the greater stressors associated with their gender and marital/societal roles [41]. Hamaideh *et al*. [18] suggested that, generally, in the Arab countries, including Jordan, women might be given a “lower status” than men, which, along with culturally derived expectations, often determines their perception of and reactions to stress. However, among students in Korea, there were no significant gender differences in gastrointestinal symptoms over the past three months [37], perhaps because the proportion of males in the Korean sample was low (<10% of the sample); and across students in Ireland, there was no association between gender and lower back pain [36].

In terms of the stressors/burdens (whether educational related and general overall), we found a positive significant relationship between each of the amount of perceived educational related and general overall stressors on the one hand, and all four most prevalent symptoms on the other. We agree with Saudi Arabia, where the most frequently occurring stressors among students were related to academic and psychological domains [42]; and a review of students’ stress reported that the sources of stress (stressors) included educational environment factors and academic factors [11]. In support, at the level of adolescent students, studies have pointed out the links between examination related stressors and somatic complaints, e.g., gastrointestinal symptoms or physical pain in Sri Lanka or Taiwan [43,44]. These features raise two points: The first is that is that stressors could also have beneficial effects, and little research considered those stressors expected to improve students’ well-being/educational process [45]. The second point is that it is useful for educators to debate ways to decrease the educational related stressors, where emphasis on formative versus summative assessments, elimination of quotas, problem-based learning, and reflective portfolios are some promising strategies [46,47].

As for the socio-demographic features, across our sample, females were associated with three of the four most prevalent symptoms, in contrast to other studies (discussed above). For age/year of study, our younger students had more sleep disorder/insomnia than their older colleagues. In Canada, the sources of stress in dental students and first-year residents varied according to their stage in the program and the period of the year [15]. It also seems that final year students (older) might have learnt ways to “harness” the stressors they encounter and turn them into positive actions for their well-being/educational process, as their long experience could had probably influenced the given stress responses compared to those students in the earlier stages of their studies [45]. For income sufficiency, we found a significant inverse relationship between income and difficulties to concentrate, (increasing income insufficiency was associated with more difficulties to concentrate), in agreement with others where the most frequently mentioned personal factors in countries where students support themselves financially were financial problems [13,48]. Specific stressors seem to differ in different parts of the world, where stressors e.g., related to fear of parents were more significant in India, whilst stressors related to students’ financial situation were more significant in western countries [11].

As regards to health behaviours/lifestyle factors, for subjective health we found that compared to students with excellent health, those with good, fair or poor health were more likely to have higher levels of three of the four most prevalent symptoms. We agree with Jordan, where both male and female students with excellent/very good health reported lower stress levels [18]. It is noteworthy to note that in contrast to the UK where students with higher alcohol consumption were more likely to report symptoms [27], smoking, alcohol or illicit drug/s use did not play a relevant role among Libyan students due to its very low prevalence in this population.

Both health awareness and quality of life had an inverse significant relationship with depressive symptoms, but not with any other symptom. Poorer quality of life and a lower level of health consciousness/ awareness among depressed students have also been described in other countries [8].

As for objective four, our results showed that the positive association between feeling burdened with a higher frequency of symptoms remained significant for three out of the fours symptoms even when controlled for gender and self-perceived health. This agrees with other research showing that stress is an independent predictor for symptoms and health complaints among university students [2,3].

The study has limitations. One variable measured each of the college related stressors and general stressor (burdens) overall, when college related stressors are many (lack of practical relevance of studies, anonymity/isolation at university, bad job prospects, problems with peers, lack of time for studies), and general stressors are many (problems with parents, friends or significant other, housing, health problems). Due to respondent burden and the study being a general health survey, we were unable to explore each variable employing many items. The study is cross sectional, no cause-effect relationships can be deduced. Females were slightly over-represented (a reality at higher education institutions in many countries). Selection bias, as students with health problems would have been less probable attend at university during the data collection is possible. Self-reports estimated the symptoms (no objective validation was conducted). Nevertheless, no valid external measurement of health complaints exists since physicians’ ratings also rely heavily on patients’ descriptions. Future research could address these limitations. The study has notable strengths. The large sample size (and representativeness), and the relatively high response rate enabled the calculation of accurate estimates across students at many Libyan higher education institutions. We mobilized many students’ demographic characteristics and health behaviour variables. No previous study had investigated in detail, the health complaints, and their associations with perceived stressors, and with students’ demographic features and health behaviours at Libyan universities.

## 5. Conclusions

The study indicates that education related and general stress play an important role in the life’s of students in Libya and that at the same time the fast majority of students is experiencing one or more health complaints. The strong relationship between perceived stressors and health complaints calls for preventive action. Stress management and relaxation programmes offered on campus may be an option to prevent stress-related symptoms. However, although it is important to help students cope with stress borne out of overall life circumstances, interventions by universities aimed at reducing the impact of academic stressors on students may also be of great importance.
